# Therapeutic potential of targeting IL-17 and IL-23 in sepsis

**DOI:** 10.1186/2001-1326-1-4

**Published:** 2012-04-27

**Authors:** Markus Bosmann, Peter A Ward

**Affiliations:** 1 Department of Pathology, University of Michigan Medical School, Ann Arbor, MI, 48109, USA; 2 Department of Pathology, The University of Michigan Medical School, 1301 Catherine Road, Ann Arbor, MI, 48109-5602, USA

## Abstract

Severe sepsis is a major concern of public health in industrialized countries. It is estimated that in the United States 200,000-400,000 cases occur annually and resulting in an extensive burden for the health care systems. To date, no FDA-approved pharmacologic agents for the treatment or prevention of human sepsis are available. The current modalities of therapy in sepsis include the standard arsenal of supportive interventions in critical care medicine and pharmacotherapy, with use of antibiotics and catecholamines. Despite such efforts, the mortality rates of sepsis have remained around 30-50 %. Extensive scientific studies have utilized animal models of disease and aimed for a better understanding of the pathophysiologic mechanisms during sepsis. Members of the IL-17 family of cytokines, as well as the functionally related IL-23, have been identified as new players in the molecular events during sepsis. Strategies for targeting these mediators with neutralizing antibodies during experimental sepsis in rodents have demonstrated efficacy, resulting in improved survival outcomes. Currently, it is not clear whether such findings can be translated to human sepsis. This review highlights the current knowledge on the biology of IL-17 isoforms and IL-23 as well as potential applications to clinical medicine.

## The IL-17 family of cytokines

The first IL-17 family member, IL-17A, was described in 1995 as a cytokine encoded by Herpes virus saimiri [1]. In humans, IL-17 was first found to be expressed by activated T cells [2]. Noteworthy, first evidence for the existence of murine IL-17 originated in 1993 under the name CTLA-8 [3]. IL-17A is a secreted and glycosylated protein with a molecular mass ~35 kDa. Several other structurally related cytokines have since been discovered. This list includes IL-17B, IL-17 C, IL-17D, IL-17E and IL-17 F. The IL-17E family member displays only 16 % sequence homology to IL-17A [4]. This is one of the reasons why IL-17E has also received the designation IL-25 in the current nomenclature system. On the other hand, a close structural relation exists for IL-17A and IL-17 F (~50 % homology, depending on species). A common structural moiety of IL-17 family members includes four conserved cysteine residues. Formation of disulfide bonds containing these cysteine residues allows for the assembly of either homodimers or heterodimers. In terms of biological activity it has been claimed that IL-17 F is 10-30-fold less potent than IL-17A, with the IL-17A/F heterodimer having an intermediate level of activity [5]. The basis for such statements has been experiments determining the levels of down-stream gene activation for chemokines such as CXCL-1 after induction by IL-17A, IL-17 F or IL-17A/F. It should be cautioned that the results of such studies may not apply to all biological functions of IL-17 family members or to all clinical situations in which IL-17 cytokines are expressed. In this report, the IL-17 family will be referred to as IL-17 s.

Noteworthy, heterodimeric IL-23 has no structural relation to IL-17 family members. IL-23 is composed of the subunits p19 and p40. Whereas the subunit p40 can also associate with p35 to form IL-12, no other binding partner for p19 has been identified so far. Thus, clinical measurements of IL-23 should preferentially be designed for specific detection of p19.

## Cellular Sources of IL-17 s and IL-23

It is widely agreed that macrophages and dendritic cells are the major cell sources contributing to the rapid release of IL-23 during disease. For example, in C57BL/6 mice after a lethal intraperitoneal injection of LPS, plasma levels of IL-23(p19) peaked as early as 3 hours [6]. In cell cultures of LPS-activated macrophages, IL-23(p19) is detectable with similar kinetics indicating very rapid induction [6].

For the IL-17 s, the situation is much more complex. Originally cloned from T cells, it is now clear that IL-17 s can be produced by a wider variety of immune cells with phenotypes other than Th17 cells. Release of IL-17 has been demonstrated from neutrophils, lymphocyte-tissue inducer cells, iNKT cells, γδ T cells and paneth cells [7,8]. There is also growing evidence that macrophages are relevant sources of both IL-17A and IL-17 F isoforms during the acute inflammatory response [6,9-11]. The capability of macrophages to induce IL-17 gene expression may depend on the origin of the macrophages, since IL-17A production after LPS was observed in murine peritoneal elicited macrophages and alveolar macrophages but not in bone marrow derived macrophages after 7 days of maturation in vitro [6].

### Biological Functions of IL-17 s and IL-23

**Figure 1:**
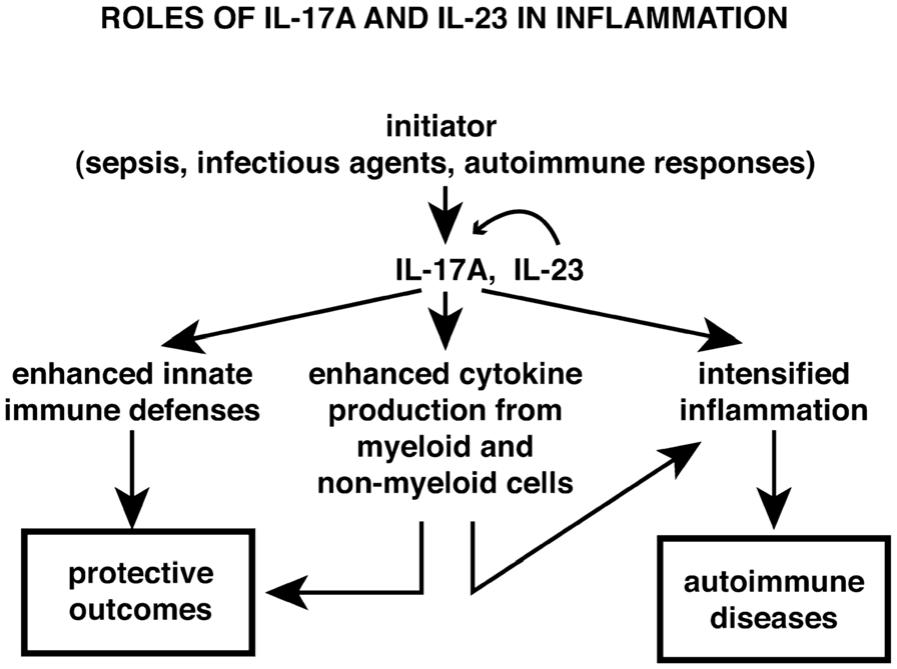
Proposed pathways related to IL-17A and IL-23 production and protective effects related to enhanced innate immunity or harmful effects due to exaggerated inflammatory responses.

There is clear evidence for the role of IL-17 s and IL-27 for the clearance of infectious pathogens. Perhaps more relevant, their role in the development of autoimmune diseases has been received wide recognition. In experimental models of rodents developing autoimmune encephalomyelitis or collagen-induced arthritis, IL-17 has been linked to the severity of inflammation in tissues [12,13]. The differentiation of naïve T cells into the Th17 phenotype has been shown to be facilitated by the presence of a cytokine milieu containing a combination of TGFβ and IL-6 [14,15]. Subsequently, as a hallmark of Th17 development, the transcription factor, RORγt, is expressed and activated [16]. Later stages of Th17 cell differentiation (clonal expansion, phenotype stabilization with IL-17 production) also rely on expression of IL-23, which is a potent inducer of IL-17 [12,13]. It is worth mentioning, that under certain circumstances IL-17 can also be produced in the absence of IL-23 and vice versa IL-23 can have biological activities independently of IL-17 [17]. IL-17 predominantly interacts with non-leukocytic cells such as epithelial cells, fibroblasts and endothelial cells but also macrophages [18]. From these cells, IL-17 initiates production of other proinflammatory mediators such as IL-1, TNFα, IL-6, IL-8, CCL20 and G-CSF, collectively resulting in an influx of neutrophils. [7,18,19]. IL-17 synergizes with other mediators such as IL-1, IL-6 and TNFα to activate tissue-infiltrating neutrophils to facilitate the effective elimination of invading bacteria or fungi. These concepts are illustrated in Figure 1.

### The IL-17/IL-23 axis and the Complement System

The complement system is an integral part of the innate immune response against infections by extracellular pathogens. Since the IL-17/IL-23 axis also directs immunologic functions towards the clearance of bacteria and fungi rather than being involved during viral infections, the roles of IL-17/IL-23 and the complement system would appear to be somewhat redundant. Therefore, it was no surprise that several interactions between these two immune defense systems are known to exist. Most notably, the complement cleavage product, C5a, at higher doses (25–100 nM) interferes with gene expression and release of IL-17 family members following LPS-activation of macrophages. Interestingly, C5a suppressed production of the potent isoform IL-17A but amplified the amounts of the weaker isoform IL-17 F [6,9]. Preliminary studies failed to observe consistent effects of C5a on production of the IL-17A/F heterodimer (Bosmann and Ward, unpublished findings). At the same time, C5a very potently antagonizes the release of IL-23(p19) from LPS-activated macrophages (by more than 80 %) [6]. In part, the effects of C5a on the IL-17/IL-23 axis can be attributed by a PI3K/Akt-dependent activation of IL-10 release. This requires ligation of C5a to the C5aR receptor but is independent of the second C5a receptor, C5L2 [6]. While it is clear that C5a drives activation or inhibition of signaling responses in macrophages via PI3K/Akt pathways, it is unknown why transcription and translation for IL-10 is enhanced whereas in the case of IL-23, there is profound suppression of its expression. At least in macrophages the RORγt transcription factor appears to be of less relevance for expression of IL-17 s as opposed to many reports describing the role of RORγt for Th17 cell differentiation and activation [6,16]. It might be noted that, in wild type macrophages stimulated with LPS, there is no measureable expression of mRNA for RORγt, whereas in IL-10^−/−^ macrophages, addition of LPS readily upregulates mRNA for RORγt [10]. We speculate that other yet unidentified intracellular factors must exist to transmit the signals of C5a on cytokine production including the IL-17/IL-23 axis. Such factors may act as transcription factors or possibly may have the biochemical composition of microRNAs.

There are abundant reports on the role of the C5a during experimental sepsis in rodents. The blockade or genetic absence of either C5aR or C5L2 improved survival rates after polymicrobial sepsis (after cecal ligation and puncture) [20]. Likewise, blockade of C5a by neutralizing antibodies reduced mortality in such models [21]. Currently, a phase I clinical trial involving healthy volunteers and using a humanized monoclonal antibody (IFX-1) against human C5a during human sepsis has been conducted in Germany. It would be interesting to determine if blockade of C5a in humans with sepsis affects the levels of IL-17 isoforms and IL-23 in plasma. Clearly, further studies in polymicrobial sepsis of rodents are necessary to define the interactions of the IL-17/IL-23 axis with C5a.

### Role of IL-17 s and IL-23 during sepsis

Blockade of IL-17A or IL-23(p19) has been demonstrated to effectively improve the outcome of endotoxic shock in C57BL/6 mice (Table 1) [6]. In this study, we compared anti-IL-17A or anti-IL-23(p19) antibodies to isotype control antibodies when administered briefly before intraperitoneal injections with LPS. Treatment with neutralizing antibody to IL-17A significantly improved survival rates from 40 % to 100 % and anti-IL-23(p19) antibody treatment from 10 % to 55 %, respectively. Another group has reported on the protective effects of anti-IL-23(p19) antibodies in a murine model of gram negative sepsis induced by *Pseudomonas aeruginosa*[22]. During human sepsis, the mRNA for IL-23(p19) in peripheral blood was elevated above levels found in healthy individuals [23]. Noteworthy, it has been suggested that dendritic cells from newborns produce higher levels of IL-23(p19) after LPS activation in vitro when compared to dendritic cells from adult donors [24]. In clear contrast, activated monocytes from premature infants (born before 29 weeks of gestation), who have an increased risk for neonatal sepsis, showed a defect in producing the common subunit p40 of IL-12 and IL-23 [25]. If such observations would also apply to p19 remains to be seen.

**Table 1:** Studies that have investigated the role of IL-17 family members and IL-23 in sepsis

**Reference**	**Species**	**Experimental Design**	**Main Results**
[26]	M. musculus	B. fragilis challenge	Neutralization of IL-17A prevented abscess formation.
[23]	H. sapiens	Expression analysis in peripheral blood	Elevation of IL-23(p19) mRNA during sepsis.
[27]	M. musculus	E. coli challenge	Neutralization of IL-17A impaired peritoneal clearance of E. coli.
[8]	M. musculus	CLP	Neutralization of IL-17A reduced mortality.
[22]	M. musculus	P. aeruginosa challenge	Neutralization of IL-23(p19) reduced mortality.
[28]	M. musculus	CLP	Production of IL-6, MIP-1α, MIP-2 by cardiomyocytes required endogenous IL-17A.
[6]	M. musculus	Endotoxemia	Neutralization of IL-17A or IL-23(p19) improved survival. Regulation of IL-17A and IL-23(p19) by C5a.
[9]	M. musculus	Endotoxemia, CLP	C5a dependency of production of IL-17 F.
[25]	H. sapiens (premature infants)	Monocytes, DCs	Defect in the production of IL-12/IL-23 p40 in premature infants.

*CLP* Cecal ligation and puncture, *DCs* Dendritic cells.

During polymicrobial sepsis after cecal ligation and puncture in C57BL/6 J mice the neutralization of IL-17A by antibody was protective by reducing bacteremia, systemic levels of proinflammatory cytokines/chemokines and improving survival [8]. γδ T cells were identified as a cellular source of IL-17A in this study. Using mice genetically deficient of IL-17A, a different conclusion was reached in terms of the role of IL-17A for survival of polymicrobial sepsis [29]. One should be aware that IL-17A deficient mice can display a compensatory hyperproduction of IL-17 F in some experimental settings such as LPS-induced acute lung injury (Bosmann and Ward, unpublished results). In part, this can be explained by the block in assembly of IL-17A/F heterodimers in the absence of IL-17A. Another point to consider is the wide variations in experimental designs of the cecal ligation and puncture model. There is no standardized consensus on the surgical protocols employed by different investigators especially including issues such as the schemes of fluid resuscitation and administration of antibiotics.

Interestingly, during both endotoxemia and CLP in mice the heterodimer IL-17A/F is expressed at 5-10-fold higher levels than either homodimer of IL-17A or IL-17 F (Bosmann and Ward, unpublished results). For the future, investigations using double-knock-out mice (IL-17A^−/−^ IL-17 F^−/−^) may provide a clearer picture, since most likely IL-17A and IL-17 F homodimers have rather redundant roles.

After challenge with live *Escherichia coli* intraperitoneally, a rapid production of IL-17A has been reported and neutralization of IL-17 resulted in a reduction of neutrophil infiltration together with impaired bacterial clearance [27]. Another report has investigated the presence of IL-17 producing T cells in the wall of abdominal abscesses following infection with Bacteroides fragilis [26]. Neutralization of IL-17 prevented abscess formation. This may suggest a potential role for IL-17 in constraining the spread of infection, presumably a critical event preceding the manifestation of sepsis.

Another hallmark of sepsis is the development of cardiac dysfunction also described as septic cardiomyopathy. We have previously shown that neutralization of IL-17A by antibody during CLP profoundly reduced the ex vivo release of proinflammatory mediators such IL-6, MIP-2 (CXCL1) and MIP-1α (CCL3) from murine cardiomyocytes [28]. Further studies will be needed to define the mechanisms by which IL-17 s and IL-23 interfere with cardiac function during sepsis, especially with respect to the situation in humans.

### Humans with genetic polymorphisms in the genes for IL-17 or IL-23

As for many genes with immune functions, several naturally occurring mutations for the IL-17 and IL-23 systems have been described [30-32]. More than 10 different single nucleotide polymorphisms in the gene for IL-23 or for the IL-23 receptor (IL-23R) have been identified [33]. For example, the IL-23R Arg381Gln mutation is associated with development of autoimmune disease [32]. Mutations in genes of the IL-17 system result in a defective immune responses to bacterial and fungal pathogens in certain clinical situations. Specifically, humans with the autosomal dominant hyper-IgE syndrome have a disability in the differentiation of Th17 cells accompanied with an impaired production of IL-17 [31]. Such individuals suffer from recurrent pulmonary infections, staphylococcal abscesses and mucocutaneous candidiasis. In other forms of chronic mucocutaneous candidiasis disease, presenting with recurrent or persistent infections with *Candida albicans*, associations with either an autosomal recessive deficiency in the IL-17 receptor A (IL-17RA) or autosomal dominant deficiency in IL-17 F have been described [30]. To date it is not known, if any of such disruptions in the genes of the IL-17/IL-23 axis are influencing the risk for the development of sepsis, probability of surviving sepsis or alterations in the immune surveillance after recovery from sepsis.

### Review

The IL-23 and IL-17 families of cytokines were originally thought to play roles in innate immune responses to contain bacterial and fungi. There is emerging evidence that these cytokines may also play adverse roles in asthma, endotoxemia and sepsis by causing exaggerated inflammatory responses. Obviously, much greater details will be required if the IL-17 family and IL-23 become potential targets for in vivo neutralization.

### Conclusions

Many basic science and clinical reports have provided an understanding of the implications of the IL-17/IL-23 axis during autoimmune diseases. There is also emerging evidence that this family of functionally related cytokines may play a distinct role during sepsis. The current knowledge justifies further investigations to evaluate the role of IL-17 family members and IL-23 during sepsis. Monoclonal antibodies to target IL-23 (e.g. ustekinumab, also cross-reacting with IL-12) have been approved for the treatment of autoimmune disease such as psoriasis [34]. Likewise, human monoclonal anti-IL-17 antibodies (e.g. secukinumab) are under development for testing in clinical trials. It remains to be seen, if such new therapeutic agents may also have a potential benefit for patients with severe sepsis.

## Competing interests

The authors declare that they have no competing interests.

## Authors Contributions and Acknowledgements

Role of the authors: MB performed all work and drafted the article. PAW supervised the work and assisted in assembly of the final report. This work was supported by grants GM-29507 and GM-61656 from the National Institutes of Health, USA, (PAW), and by the Deutsche Forschungsgemeinschaft (Project 571701, BO 3482/1-1) (MB). We cordially thank Beverly Schumann and Sue Scott for assistance in the preparation of the manuscript. All authors read and approved the final manuscript.
